# ‘Really putting a different slant on my use of a glass of wine’: patient perspectives on integrating alcohol into Structured Medication Reviews in general practice

**DOI:** 10.1080/16066359.2023.2207017

**Published:** 2023-04-28

**Authors:** Mary Madden, Duncan Stewart, Jim Mc Cambridge

**Affiliations:** a Department of Health Sciences, University of York, York, UK; b Centre for Primary Health and Social Care, School of Social Professions, London Metropolitan University Department, London, UK

**Keywords:** Alcohol, clinical pharmacy, medicines review, primary health care, brief intervention

## Abstract

**Background:**

Alcohol is often overlooked in primary care even though it has wide-ranging impacts. The Structured Medication Review (SMR) in England is a new ‘holistic’ service designed to tackle problematic polypharmacy, delivered by clinical pharmacists in a general practice setting. Implementation has been protracted owing to the COVID-19 pandemic. This study explores early patient experiences of the SMR and views on the acceptability of integrating clinical attention to alcohol as another drug linked to their conditions and medicines, rather than as a standalone ‘healthy living’ or ‘lifestyle’ question.

**Method:**

Semi-structured interviews with a sample of 10 patients who drank alcohol twice or more each week, recruited to the study by five clinical pharmacists during routine SMR delivery.

**Results:**

SMRs received were remote, brief, and paid scant attention to alcohol. Interviewees were interested in the possibility of receiving integrated attention to alcohol within a SMR that was similar to the service specification. They saw alcohol inclusion as congruent with the aims of a holistic medicines review linked to their medical history. For some, considering alcohol as a drug impacting on their medications and the conditions for which they were prescribed, introduced a new frame for thinking about their own drinking.

**Conclusions:**

Including alcohol in SMRs and changing the framing of alcohol away from a brief check with little meaningful scope for discussion, toward being fully integrated within the consultation, was welcomed as a concept by participants in this study. This was not their current medication review experience.

## Introduction

Alcohol is an addictive psychoactive drug (ethanol) (Kypri and McCambridge 2018), which is a major contributor to the burden of disease, including long-term non-communicable diseases (Rehm et al. 2017; Griswold et al. 2018). Although heavy drinkers are most at risk from the consequences of alcohol consumption, all drinkers are at risk of alcohol harms, as are nondrinkers around them (Babor et al. 2010; Wood et al. 2018). Alcohol is often overlooked in primary care although it has wide-ranging impacts on health and wellbeing and affects the way people take their medicines, their safety and effectiveness, even at what may seem low doses (Stewart and McCambridge 2019; McCambridge and Stewart 2020). It is specifically overlooked within medication reviews (McCaig et al. 2011; Morris et al. 2019).

Many people are living with multiple, often long-term, medical problems for which medications are prescribed which increase treatment burden and decrease overall quality of life (Academy of Medical Sciences 2018). In addition to direct harms to health, alcohol, even at relatively low levels of consumption, can interact with and counter the effects of medications and exacerbate symptoms, contributing to a ‘prescribing cascade’, especially if it causes new symptoms or exacerbates adverse drug reactions which are misinterpreted as a new condition (Rochon and Gurwitz 2017). Consensus on what constitutes an alcohol-interactive medicine is lacking, and little is known about how people conceptualize the risks posed to their health from concurrent alcohol and medicines use (Madden, Morris, Stewart, et al. 2019).

In the UK, a new Structured Medication Review (SMR) service is a key element of the response to problematic polypharmacy in primary care (DHSC Medicines Directorate 2021). SMRs are delivered by appropriate health professionals, mostly a greatly expanded clinical pharmacy workforce recruited to General Practitioner (GP) practices experiencing workforce shortages (Mills et al. 2022). The SMR was introduced in England during the COVID-19 pandemic, which protracted its implementation at practice level (Stewart, Madden, et al. 2021; Madden et al. 2022). Nationally determined contract performance indicators were suspended during this time, meaning general practices, operating within newly forming Primary Care Networks (PCNs), were determining their own practice-specific targets based on clinical pharmacist capacity (Madden et al. 2022).

The SMR service specification described an invited, personalized, holistic review of all medicines for people at risk of harm or medicine-related problems (e.g. those taking multiple medications, or medication that is potentially addictive or commonly associated with medication errors) (NHS England 2021). SMRs are expected to last 30 min or more to allow for shared decision-making (NHS England 2021). The risks from alcohol interactions with medicines are recognized in the specification, raising the prospect of including attention to alcohol (and other public health behavioral risk factors), but without providing guidance on how to integrate this within the review (NHS England 2021). SMR practitioners are encouraged to review alcohol use and deliver brief advice. Links are provided to the UK Chief Medical Officers’ low risk guidelines; the AUDIT C tool; a National Health Service (NHS) structured advice tool and a free Health Education England e-learning course. SMR practitioners are also encouraged to implement a range of other public health brief advice interventions for smoking, falls and frailty, physical activity and weight management, with links provided to online resources and training materials for each (NHS England 2021).

A study of early implementation practices reported by clinical pharmacists showed SMRs did not match the idea presented in policy documents of an invited, holistic, shared decision-making opportunity offered by prescribers experienced in history taking (Madden et al. 2022). Patient perspectives of such medication review innovations are under-researched. What there is in the pharmacy literature shows a gap between the person-centred rhetoric of pharmacist-led medication reviews and actual practice (Latif et al. 2013; Madden, Morris, Stewart, et al. 2021; Madden et al. 2022). In the large alcohol brief intervention literature, relatively little attention has been paid to patient receptivity, despite significant difficulties with implementation in primary care (McCambridge and Saitz 2017; Kaner et al. 2018; Madden, Morris, Ogden, et al. 2021). Willingness to discuss alcohol with a health professional may count for little if patients do not see the relevance to their own health situation and goals (Madden, Morris, Ogden, et al. 2021).

This study forms part of a research programme on the inclusion of alcohol within medication reviews as a legitimate subject for discussion with pharmacists (Madden, Morris, Ogden, et al. 2021; McCambridge et al. 2021). Findings from previous studies show pharmacists are not confident with the subject of alcohol, do not feel it is their job to address alcohol issues beyond giving consumption advice, and are worried about how to deal with any difficulties that might arise if they open up discussion (Morris et al. 2019; Madden, Morris, Stewart, et al. 2021). A decision to minimize attention to alcohol in medicines reviews may therefore stem from pharmacists rather than patients.

Rather than asking pharmacists to take on a new public health role (Atkin et al. 2021), the proposed new Medicines and Alcohol (MAC) approach locates alcohol within the core pharmaceutical role, providing clarity to pharmacists and patients currently unsure about the place of alcohol conversations (Madden, Morris, Stewart, et al. 2019; Madden, Morris, Atkin, et al. 2020; McCambridge 2021). Study objectives here were exploratory, to investigate in depth early patient experiences of the new SMR service as currently delivered in routine practice, their views on the acceptability of discussing drinking alcohol with health professionals and on using the proposed MAC approach within the SMR service specifically. The aim was to understand barriers and opportunities for the discussion of alcohol in SMRs from the patient perspective and explore the potential for, and receptivity to, the innovation involved in incorporation of the MAC approach, i.e. linking alcohol to medications and conditions within SMRs. It is one of a number of studies seeking to understand pharmacist medication review practice and skills as a potential site for intervention (Dhital et al. 2015; Stewart et al. 2020; Madden, Morris, Stewart, et al. 2021) and find better ways of managing alcohol in general practice (McCambridge and Rollnick 2014; McCambridge and Saitz 2017; McCambridge and Stewart 2020).

## Methods

The study received NHS Health Research Authority approval (REC reference 20/HRA/1482). Subjects have given their written informed consent. Patients were recruited by five clinical pharmacists working at different practices within one Primary Care Network in Northern England during routine SMR delivery. Consecutive SMR patients were asked by the pharmacists if they would be interested in taking part in a study about how health and wellbeing are discussed in medication reviews. If patients accepted, a brief screening form was completed, including a single item alcohol screening question embedded in a range of other health and service utilization questions: ‘How often do you have a drink containing alcohol?’ Patients were eligible if they consumed alcohol at least twice per week. This screener was adopted following a validation study (Stewart et al. 2021). Eligible patients were then provided with a study information statement and completed a consent form.

Ten semi-structured interviews were conducted by telephone by the lead author, including one video call. Recruitment to SMRs was slow and all eligible patients identified during the study time frame who agreed to participate were interviewed. Sample demographics and medications taken (1–8 per person; mean = 4) are detailed in Table 1. Topic guides and interview structure (see Supplementary Appendix 1) were informed by previous experience of interviewing sensitively on alcohol and medication use with input from a patient advisory group (Madden, Morris, Atkin, et al. 2020; Madden, Morris, Ogden, et al. 2020). Interviews lasted 30–70 min (mean = 46.5). Audio-recordings were professionally transcribed verbatim. A modified framework method (Gale et al. 2013) supported a constructionist the-matic analysis (Braun and Clarke 2006). Interview transcripts were coded in NVivo (version 12) to produce a list of initial descriptive themes identifying alcohol and medication practices, SMR experience, views on discussing alcohol with health professionals and on the inclusion of alcohol framed as a drug. The topic guide formed the initial coding framework. Comparative analysis identified common, recurring, and conflicting perspectives, and noted the ways in which accounts were constructed. The analysis was undertaken by the first author and developed iteratively with the full author team.

## Results

### Received SMR content and duration

SMRs were delivered remotely in ways which departed considerably from the policy vision for the new service. None of the patients in this study were invited to participate as part of a distinct service. 8/10 appointments were initiated as a repeat prescription or annual medication review, flagged when ordering medication. The other two patients (2 & 3) requested an appointment with a GP to discuss the effectiveness of their medications and were referred to a pharmacist. One patient (5) thought the review received was with an unfamiliar GP rather than a clinical pharmacist. All reviews were undertaken by phone, as was occurring elsewhere at the time (Madden et al. 2022). Three would have preferred a face-to-face appointment had they been given the option (3, 5, 6).

None of the patients had prepared for the review other than by assembling their medications. One, who had been expecting a short appointment remembered it lasting for 30 min:

… it seemed very thorough … I’ve had these reviews before … with the GP … I expected it to be literally a two-minute job … you’re on this, you’re on that, is everything fine, and I say yeah, they say right, okay, box ticked and that’s it (1).

This review was an exception in duration. Four patients said the review lasted 5 min or less; one 5–10 min; two 10– 15 min; one 15–20 min and one 20 min. These estimates included the time taken to recruit the person into this study as part of the review.

Interviewees struggled to recall the content of their review other than they were asked questions in a polite and friendly manner. This was also the case in the 30-minute appointment:

It’s difficult to remember because … it was just a phone call for me, just to get … the box ticked on my prescription. So it’s not something that I really set out to remember (1).

For some patients, the review was considered useful for getting access to medication (7, 8, 5), a GP appointment (2, 3, 10) or ‘general reassurance’ (1). Other than this they were left unsure of its relevance to them:

I would say it was ticking the box type of exercise quite honestly, we’ve got to do it … done and there we go … I wouldn’t say it was tailored to me at all … I would say they could have done without it quite honestly (8).

### Inclusion (and exclusion) of alcohol in the review

Clinical pharmacists who asked about alcohol did so as a quick question and answer transaction, gathering information about amounts consumed without linking this to issues relevant to the patient’s medication or conditions. Two patients said alcohol was not raised at all in their review. Two others could not remember but assumed they had been asked as part of their recruitment to this study. The remaining six patients remembered being asked how much they drank but with little discussion, other than to ask about taking part in this study:

Asked me how many units I probably drink in a week, then I explained, I said I’ve cut down … he basically said it was quite moderate … I was honest about it, but he didn’t really go into any details … like advising or anything … it was just like for the computer or whatever (2).

This patient was taking the antidepressant Citalopram. In her interview she explained that she drank wine and cider at the weekend to help her relax, she was aware she was using alcohol to manage her symptoms and had been cutting down because:

… it is advised not to drink on the medication … so it makes me think twice about doing so … some nights I’d get two bottles of wine, but now … it would just be the one … through the week as well sometimes … I’d do it every other day … but now I have to think about it a lot more and it puts me off doing it (2).

Advice to avoid drinking whilst on the medication given by her therapist was not reinforced by the clinical pharmacist in her review:

… they [therapist] say … it is classed as like a depressant, alcohol, so you might feel really good one bit, but then … it can bring your mood down, so it counteracts it a little bit. So I understand in that sense that it could make the medication not work and make symptoms worse from anxiety and panic and stuff (2).

This was one of two patients who said during course of their interviews for this study that they would like to change their drinking, but had not mentioned this in their review with the clinical pharmacist:

… sometimes I don’t know when to stop, so if it is in the house, I would continue, so I’d like to know to stop when I wanted to, or … not when I wanted to, to … put a limit on myself … (2).

Another patient was concerned about the efficacy of his antidepressant Sertraline, which can cause gastrointestinal side effects, and the amount of medication he was taking for gastric reflux. He drank eight pints of beer each night over the weekend to help him relax. He knew he was drinking more than was recommended, so was unsurprised to be advised so by the clinical pharmacist:

I think he said it was a bit high, so that was about it … Heard it before sort of thing … Yeah, if you’re having eight pints a night it’s obviously too much, isn’t it … (3).

He had previously been advised that the amount of alcohol he was consuming was over recommended limits and this knowledge did not impact on his drinking, he had ‘heard it before’. The possible role of alcohol in relation to his specific concerns about efficacy of antidepressants and causes of gastric irritation was not discussed in the review.

An interviewee taking Naproxen for pain following a hip replacement and a subsequent fall, said he was unsure how to make sense of the units of alcohol referred to in his review but did not raise this with the clinical pharmacist:

I don’t know how much 14 units is to tell you the truth [UK Chief Medical Officers recommendation of 112g alcohol spread over one week] … we didn’t get into how many units is in a pint or in a half or in a glass of wine or anything else like that (4).

Naproxen is a non-steroidal anti-inflammatory drug which increases the risk of gastro-intestinal hemorrhage. This risk is increased further by alcohol, but the alcohol focus in his review was again on advising on generic weekly units rather than in connection with the specific risks of stomach bleeding and further falls.

One patient drank large measures of whiskey at the week-end and the focus in his review was also on amounts consumed rather than its impact on the efficacy of the cardiovascular, diabetes and gastro protective medication he was taking (6). Another patient taking an antidepressant that carried an alcohol warning said she did not talk about this in her review, she expected the pharmacist to say something only if she was, ‘falling down drunk every day’ (7). When initially asked, most interviewees, including those, like this woman, who had seen warnings on their medication, did not think there was any particular link between their medications or conditions and their own drinking. As in previous studies, patient perceptions of personal health hazards from alcohol were mostly focused on stereotypical conceptions of alcohol dependence rather than how it impacted on their own health in other ways. During the course of the interview, some, including her, began to make connections that, ‘… really put […] a different slant on my use of a glass of wine’ (7):

… you’ve made me think actually, about when I do have a drink and this falling to sleep [on a night out] is anything to do with my medication, I’ve not thought of it like that (7).

One exception was a patient drinking 30–40 units a week who readily made connections between his medication and alcohol throughout the interview (8). This, however, and another thread on previous investigations of liver function, was not picked up as part of his review. He did not recall the subject of alcohol coming up other than to refer him to this study. Having suffered persistent reflux and throat symptoms he had stopped taking Lansoprazole to reduce stomach acid several years ago because he did not feel it was working. He was currently trying Candesartan to treat high blood pressure rather than Ramipril for his hypertension:

I’ve never been sure if it was booze, alcohol or the Ramipril so I moved onto Candesartan … I do think that, you know, alcohol … might be exacerbating my throat problems because, obviously, it’s an irritant, isn’t it? … I also think that, obviously, alcohol probably affects my blood pressure as well (8).

This interviewee had been diagnosed with Gilbert syndrome, a largely benign condition which can cause jaundice, and nonalcoholic fatty liver disease which he said had, ‘removed a bit of the worry’ about his drinking (8). This may be because genetic problems with liver enzymes could help him explain abnormal liver function without implicating alcohol. He was also reassured to receive a low score on a liver fibroscan:

I’m just going to continue as I was and … live my life. I never wanted to give up drinking because … it is my social life (8).

However, when asked, he also spoke about the downsides of his drinking. Alcohol disrupted his sleep, and he was concerned to prevent his drinking from getting heavier as he got older:

… I could probably do with cutting down a bit … it would be quite easy … to start slipping into heavier drinking … I don’t want to be … an all-day drinker sat in [name of pub chain] … I think I could very easily fall into that routine and it’s not particularly something I want to do … I’d like to be able to not fill the day with drink (8).

Another patient drinking 30-35 units a week and taking medication for cardiovascular disease, an antidepressant and Lansoprazole to reduce stomach acid said he, ‘would be guessing’ when asked to recall any discussion of alcohol in his review (1). He was happy with his drinking although it caused arguments with his wife and sometimes fueled his anxiety:

If I was to pack in drinking I’d probably find things easier mentally I think. But I enjoy the social aspect of it. I think at the minute I’ve got the balance about right. My wife will tell you different, she will say that I do drink too much, and she’s probably right (1).

### Acceptability of talking to health professionals about alcohol

Interviewees recognized that alcohol consumption could be a sensitive topic and that some drinking behavior, specifically binge drinking and alcohol dependence, carried stigma which they wanted to avoid. They were open to finding out if alcohol was impacting on their treatment and health. Two said they found sexual health and weight more difficult subjects to talk about (7 & 8). All said they were willing to talk to health professionals about alcohol, including pharmacists. However, rationales for drinking, its downsides and using alcohol for symptom management discussed in the interview were not at all a feature of their consultations in primary care, with any health professional. Six patients said their usual experience was limited to providing quick answers to questions about how much they drank. The other four spoke in more detail about when and why they had been asked about drinking and their thoughts on what was said. This included the man diagnosed with Gilbert syndrome who had spoken to a number of GPs and consultants about his liver and the woman on Citalopram who contrasted the advice she was given from her therapist with the usual enquiries she experienced from health professionals, ‘for the computer’ (2):

… she was actually explaining … she was giving me advice as well instead of just asking questions and telling me that it does act as a depressant … (2).

A man who, fourteen years ago, had an operation to remove a calcified pericardium, was trying to reduce his drinking to the recommended 14 units per week (9). His anticoagulant medication had been changed from Warfarin to Apixaban to reduce the increased risk of bleeding from alcohol and he remembered the nurse telling him this meant he could ‘drink that bit more’:

… with Apixaban you don’t have to be so hard on yourself cutting down on your drink. You can drink that bit more … She also added that she shouldn’t have told me that anyway (9).

‘Shouldn’t’, perhaps because this framing implies protecting alcohol consumption from the effect of the medication rather than the other way around. He said he was willing to take advice where necessary:

… if your doctor turned around tomorrow and said that’s it … you’ve got to pack it in, that’s me, I’ll be finished … before I had my heart operation … they said … how do you feel about stopping drinking? I said, fair enough, that’s it, I’m done. So, he said … don’t you want to a cooling off period? I said, I’m not an alcoholic, I don’t need a cooling off period. I said, you’ve told me to try and do it, so I’m doing it as of this minute. And I did (9).

Perceived personal distance from the stigmatized idea of an alcohol problem may have helped him be confident in his own ability to cease drinking.

The fourth patient recalled his resistance to having been told by a GP to drink within the recommended limits. He was taking medication for cardiovascular disease, Lansoprazole to reduce stomach acid, an antidepressant and drinking 30–35 units of alcohol per week:

I’ve gone away thinking she’s saying I should be drinking only 14 units a week, I’m breaking a rule there. But you sort of scrub it under the table … and just forget about it … It seems unrealistic to me … I don’t really know that many people that do (1).

### Acceptability of paying clinical attention to alcohol within the medication review

All interviewees thought the MAC approach of including alcohol as another drug in medication reviews was a good idea and most thought that they would benefit personally from this (see Table 2 for direct quotations from all ten). One said this approach would have been more relevant to him before he had reduced his drinking (5). One was worried about the prospect of being told not to drink and was unsure about the benefit of changing the habit of a lifetime at the age of 75 (10). He was drinking 36–39 units per week and could not recall being asked about alcohol in his review. He took medication for cardiovascular disease, was recovering from bowel cancer and had recently been investigated for a kidney problem after collapsing in his bedroom.

The idea of the SMR as the 30-minute, invited, holistic medication review described in national policy required some explanation, because this was not the interviewees’ experience of the review they received and most other health care appointments they experienced were brief and focused on one aspect of their health. One interviewee who had been asked the same questions repeatedly by different health professionals who did not know his medical history, said the proposed alcohol inclusive version of the SMR should not be ‘standalone’ and should be informed by medical records (8). These suggestions are already included in the SMR specification.

## Discussion

In keeping with our study of early SMR implementation, SMRs received by these interviewees did not match the ideal for patients presented in policy documents but were remote, brief and focused on fulfilling routine medicines-related tasks in response to backlogs (Madden et al. 2022). Rather than being invited to take part in a new service for which they could prepare, patient or practice-initiated routine medication enquiries and reviews were categorized as SMRs if patients receiving these fitted any SMR target group criteria. As in previous studies of pharmacist medication review practice, even when questions about alcohol were included, the consultation afforded little space for patients to raise concerns relating to drinking alcohol (Morris et al. 2019; Atkin et al. 2021; Madden, Morris, Stewart, et al. 2021). Here, that included highly relevant clinical issues related to medicines for cardiovascular disease and diabetes, drugs which increased risk of stomach bleeding and antidepressants which impacted on the central nervous system (Madden, Morris, Stewart, et al. 2019; Morris et al. 2019). Where alcohol was included at all, this was as a cursory check of units consumed, sometimes with minimal information given on recommended units, without consideration of the specific implications of taking this drug in combination with others. Important opportunities for intervention were therefore missed (Morris et al. 2019; Madden et al. 2023a).

In terms of their own drinking, findings here echoed those of our previous research in that people distanced themselves from the stigmatized idea of an alcohol problem, were defensive about being told not to drink, found recommended units hard to calibrate with their own drinking, and were skeptical about such recommendations if they conflicted with what they regarded as ‘normal’ (Quirk et al. 2016; Madden, Morris, Atkin, et al. 2020; Gough et al. 2020; Madden, Morris, Stewart, et al. 2020). As in these studies, interviewees began by discussing alcohol as another part of life in isolation from medicines health and illness, but when asked about the MAC approach of linking alcohol to medications and conditions, interviewees began to make their own connections between their drinking and health.

The SMR service is designed to tackle potential harms from polypharmacy and the burden for patients and health professionals of managing multiple treatments for long-term conditions. Interviewees were interested in receiving information on how medications interacted with alcohol and how this might affect their health, in order to make better informed choices, as long as this was done sensitively (Madden, Morris, Atkin, et al. 2020; Madden, Morris, Ogden, et al. 2021). The prospect of changing the framing of alcohol in SMRs away from a decontextualized alcohol enquiry, to integrate attention to use of this drug alongside consideration of medicines and the conditions for which these are taken, was strongly welcomed by participants in this study. Despite national and international recommendations that alcohol screening and brief interventions should be routinely delivered in primary care settings, many at-risk drinkers remain unaware of how alcohol consumption might be contributing to current or future ill health (Rosário et al. 2021). Making these links salient in medication reviews could go some way toward communicating the breadth and nature of the risks posed by alcohol consumption and challenging the view that alcohol only poses a problem for an extreme, stigmatized minority (Burton and Sheron 2018; Room 2005).

The COVID-19 pandemic placed limitations on clinical pharmacists’ capacity for patient-facing work and for data collection in primary care. This small, exploratory study nonetheless produced new and richly textured data, providing insights into early patient experience of SMRs and the acceptability of including alcohol as another drug in the medicines review consultation. By virtue of their participation in a study about how health and wellbeing are discussed in medication reviews, these patients may be more open to talking about alcohol than others. Pharmacists who recruited patients to the study were aware that the focus was alcohol but, despite this, their SMR consultations provided little opportunity for patients to discuss the subject. Slow recruitment to SMRs during the pandemic and consequential sampling constraints inhibited purposive sampling and preclude any claims about data saturation. The resulting sample, while modest and lacking demographic diversity, nevertheless provides a useful pragmatic snapshot of early SMR practice during the pandemic from a patient perspective.

Adapting SMRs to routine practices and remote working in pressurized GP practices may be setting unhelpful precedents for future SMR conduct (Madden et al. 2022), and there is an opportunity cost of SMR implementation without prior adequate skills development, testing, and refinement in this setting (Atkin et al. 2021; Madden et al. 2023b; Wright 2016). A sister study exploring clinical pharmacists’ experiences of discussing alcohol with patients in their new clinical role in GP practices found a lack of confidence and training in the subject; when it was raised at all, enquiries about alcohol in medicines reviews were focused on particularly heavy drinking and calculating dose and level of consumption, leading to crude advice to reduce drinking (Madden et al. 2023a). Pharmacists readily acknowledged they were not regarding alcohol as a pharmacologically active drug in their pharmaceutical practice, and that this was an obvious limitation; like the patients in this study, they were interested in learning more about incorporating it into reviews in this way (Madden et al. 2023a). Across these studies it is clear that the contexts affecting general practice are evolving rapidly, with profound implications for patient care more broadly, and attention to alcohol in particular.

More research is needed to understand how health professionals can initiate and conduct conversations about alcohol in routine practice within this rapidly changing environment, in ways that will successfully engage patients in a clinical context. Alcohol is not pharmacologically inert and clinical pharmacist expertise in medicines can provide role legitimacy to discuss alcohol, i.e. the drug ethanol, in relation to the safety and effectiveness of medicines. The rationale for asking patients about alcohol should be clear so that people know why they are being asked and how it can contribute to making the consultation helpful to them.

## Supplementary Material

Supplementary: Interview Guide

## Figures and Tables

**Table 1:** Self-described demographics and medications.

	Work status	Age	Age left school	Highest qual.	Gender identity	Sexuality	Nationality/ethnicity	Medicines
1	Full time	61	18	Higher National Diploma	M	Het	White British	Sertraline (20+ years) Atorvastatin Aspirin Lansoprazole
2	Part time	27	17	General Certificate of Secondary Education	W	Het	White British	Citalopram (10 months)
3	Full time	41	16	Undergrad degree	M	Het	White British	Lansoprazole Sertraline (10 weeks)
4	Full time	54	16	City and Guilds vocational	M	Het	White British	Lansoprazole Naproxen
5	Retired	79	15	None	M	Het	White British	Eye drops Paracetamol Aspirin Mebeverine Lansoprazole Docusate Laxido
6	Retired	73	15	None	M	Het	White British	Montelukast Simvastatin Tildiem Furosemide Metformin Brown and blue inhalers Lansoprazole
7	Semi-retired	58	18	Business and Tech. Education Council Diploma	W	Het	White British	Two inhalers Fexofenadine Fluoxetine (for many years) Oxytetracycline
8	Full time	54	18	Advanced level of the General Certificate of Education	M	Gay	White British	Candesartan
9	Retired	75	15	None	M	Het	White Scottish	Apixoban Adesan Simvastatin Fostair inhaler Vitamin C Cod liver oil
10	Retired	75	15	Technical college	M	Het	White British	Loperamide Lansoprazole Atorvastatin Amlodipine Clopidogrel Bisoprolol

**Table 2:** Acceptability to patients of considering alcohol clinically within the medication review.

Patient	Quote
1	… I can certainly see the obvious benefit from that … you’d want to know if what you were doing socially was negating the effect of the medication for a start … At my level [of drinking] I think I would benefit from that, at least the knowledge of what alcohol intake might or might not be doing to me … I have got this heart disease … I’m fitter than a lot of people our age … I think I am, and [my wife] says, yeah, but it’s on the inside.
2	… it would make people think about it twice and not just brush it off like as if it is a checklist … deeper thinking about it … it’s a lot of stigma around it [now] where it’s just alcohol rather than being treated like a drug … there’s people around me that notice that if I do have alcohol in the house, I won’t save it for another day … I’d drink it all in the one night like a binge drink … I don’t know why I do it, but I’d like to understand why I do that.
3	I think it’s a good idea … to go into that detail … the effect of it, because I guess most people … well, I’m generalizing here, but there will be a lot of people that … drink over the recommended limit, they know you’re drinking too much … I think that would just be a lot better idea … look at bit more deeply into the person … take some pressure off the GPs as well, wouldn’t it, I suppose.
4	I think it’s probably a good idea, because … your drinking, it could have a knock-on effect, it could nullify some of your medications … I think it’s the stigma sometimes … you get tarnished with … you’re a bit of an alcoholic or you’re a bit of a binge drinker because you have a lot of pints at a weekend … you mentioned earlier about alcohol having ethanol in it. Now, I don’t think a lot of people know that … I think you need to make people more aware … that it’s got this ethanol in it, then …, it can have a knock-on effect of the medication they’re actually taking … It just needs to be made a bit more clearer to people.
5	[My wife and I] … we’re aware that if you take excessive alcohol or … more than two drinks a night it can affect the medication you are on … I don’t think what we’re drinking at the moment is out of order … many years ago … we carried on drinking until we went to bed … in the past, it may have well been [a gastric irritant] … I can see it could have a bearing on it and it might have been one of my problems early days because … a bottle of whiskey would not be out of the question some nights … it’s common sense when you stop to think that it can react on your tablets …
6	… the common people don’t know what’s happening when they’re taking a certain drug and also having a drink. So if somebody turned round to you and said, you shouldn’t drink any more than this amount or you shouldn’t drink at all while you’re taking this, you should be listening … I have no clue at all, really [how it is interacting with my medication] and I would like to know.
7	… you’ve made me think actually, about when I do have a drink and this falling to sleep is anything to do with my [antidepressant] medication, I’ve not thought of it like that … I think that would be a good idea, I would certainly be open to it. This is really putting a different slant on my use of a glass of wine, doesn’t it? … So, that holistic approach would be really good … and the effects that alcohol might be having.
8	Sounds okay in theory, but how it works in practice … I wouldn’t want it to be standalone … you mentioned they’d have access to my records …. So, if the pharmacist has looked at what’s been asked before … And has further questions, comments, that’s okay. … I think you can only go ahead with it if it’s properly resourced and … doesn’t just come down to another tick box … if you go once, you think … they obviously haven’t looked up … previous history then, obviously, you’re going to not want to continue, are you? … I would definitely go at least once or twice … I think it sounds ideal … if I can go and someone can pull all those strands together … I’m surprised it’s not already happening … the last time the gastroenterologist called me in for review … they hadn’t reviewed anything … they haven’t even opened the file … there was certainly no consideration of anything else that was going off with my health …
9	I think it would be all for the good … I’d be very comfortable with that … just because you drink doesn’t make you an alcoholic … but you could be on medication and maybe too much alcohol is not good for you … if they say, that’s it [own name] I will say, that’s fine. I can still go in the pub and I can drink my [brand name] 00, which is alcohol free and it’s not a bad drink by the way, if ever you care to try it.
10	I haven’t got a problem with it … Well, as long as they didn’t stop me drinking altogether … it’s something I’ve done all my life … if they said I’ve got to stop, I suppose I’d have to stop … I’m 75 now, so it’s not going to really affect me all that much, is it, for the rest of my life?

## Data Availability

Research data are not publicly available because this could breach anonymity and ethical consent was not obtained to do so.
