# Supplementary material for: ‘Really putting a different slant on my use of a glass of wine’: patient perspectives on integrating alcohol into Structured Medication Reviews in general practice
Source: Addict Res Theory. Author manuscript; Available in PMC 2024 Mar 8. (PMC7615723; doi:10.1080/16066359.2023.2207017)
Supplement: Supplementary: Interview Guide [file EMS194525-supplement-Supplementary__Interview_Guide.docx]

**CHAMP-1 SMR PATIENT PERSPECTIVES INTERVIEW GUIDE**

**Introduce self and interview**

**Background (understand a bit more about you and your health)**

We’re talking today because you’ve had a medication review with the pharmacist, can I ask you a bit about the meds you are taking? Do you have many to take? Have you been taking them for a long time? (would you mind telling me a bit about the health conditions you take them for?)

- Use of **medication** –
  - How do you fit these into everyday life – talk me through your routine – Is this easy enough to manage? Disruptive?
    - Any recent changes?
    - Any worries about taking any of them?
    - Ever had an actual problem caused my medication?
    - Ever given up on any meds you thought weren’t working? [if so did you seek advice first? from whom?]
- Ask you a bit about drinking - Can you tell me a bit about how **drinking** fits into everyday life?
  - When do you drink? What do you generally drink? Where? With whom?
    - Did the pandemic make a difference?
    - What do you enjoy about drinking?
    - How has your drinking changed from when you were younger?
    - Are there any downsides to drinking for you?
- How does **drinking fit in with your medication**? Do any meds alter the way you drink?
  - - Ever skipped meds if you are drinking. Why? Consequences?
    - Drinking ever increased or decreased the effects of your medications?
    - Drinking ever affected your ability to take meds – e.g. forget?
- Do you ever use **drinking** to help with symptoms e.g. to get to sleep? Manage pain? Relax? Other ways?
- Does drinking affect any of your conditions in particular?
- Overall, how would you describe your own drinking? Is there anything you would like to change or not?

Thanks. Are we Ok to move on to talking about the SMR you had?

**Experience of the SMR**

- How you were invited for an SMR. Why? (what is it for?)
- Mode in which conducted (telephone/video/in person - any choice offered?) How they found this
- Did you do anything to prepare before the appointment?
- Recall of the actual review
  - Can you walk me through what happened
  - Did you agree what was to be covered together? (Who set the agenda? Any concerns?)
  - Did you ask questions about your meds and health? Were these answered?
  - Have you had a medicines review before? Who with? How did this compare?
- Experience of conversation overall
  - Was it useful to you? - what and why
  - What about how the pharmacist conducted it- what they did well/not well, why liked or disliked
  - Feel listened to and being heard/understood
  - Tailored to you/generic
  - Length of time and whether good pace/hurried
  - Any changes made since review (reflections on this- feel involved in the decision- follow up? Who with – GP? Social prescribing link worker?)
  - Have you got any suggestions to improve SMR and/or interaction with pharmacist
- Discussion of alcohol
  - Recall any? How (when) it was introduced; (how it went)
  - How did you feel about it being raised and discussed– impact on feelings - what and why
  - Usefulness- what and why
  - Comparison to previous alcohol discussions with health professionals (GP, nurse, social prescriber) (if any)
- Anything else you’d like to say about the SMR?

**Role of PCN clinical pharmacist at the GP surgery**

- Had you spoken to the pharmacist who did SMR before? Involved in your care? How did you find them? Did they explain their role? Aware of clinical pharmacists at GP?
- Thoughts on how this role fits with GP and community pharmacist
- Potential for the clinical pharmacist role to be useful in treatment of their own condition (mgt of meds) and their health generally?
- Changes experienced in primary care due to pandemic (awareness of primary care networks and how things are changing)

Finally: **Thoughts about how alcohol might be discussed in SMRs**

If we can pick up the alcohol thread of our discussion again

- What do you think about including drinking as a subject for discussion with a clinical pharmacist in SMR? (how does this compare with talking about it with GP or other health profs?)
- Do you think there would be any benefits to you in discussing alcohol in SMRs?
- What about concerns about how alcohol (another drug) could be impacting on your conditions or interacting with medication?
- Any thoughts about how your own drinking could be discussed comfortably in SMRs (what would work for you)

**Anything that they wish to add**

**Background Information** (demographics)

**Close**
